# ﻿*Amentotaxus×hybridia* (Taxaceae), a new natural *Amentotaxus* hybrid from southeast Yunnan province, China

**DOI:** 10.3897/phytokeys.226.103005

**Published:** 2023-05-23

**Authors:** Lian-Ming Gao, Gui-Liang Zhang, Zhi-Qiong Mo, Philip Thomas

**Affiliations:** 1 CAS Key Laboratory for Plant Diversity and Biogeography of East Asia, Kunming Institute of Botany, Chinese Academy of Sciences, Kunming 650201, China CAS Key Laboratory for Plant Diversity and Biogeography of East Asia, Kunming Institute of Botany, Chinese Academy of Sciences Kunming China; 2 Lijiang Forest Diversity National Observation and Research Station, Kunming Institute of Botany, Chinese Academy of Sciences, Lijiang 674100, Yunnan, China Lijiang Forest Diversity National Observation and Research Station, Kunming Institute of Botany, Chinese Academy of Sciences Lijiang China; 3 Hekou Branch of Administration Bureau of Daweishan National Nature Reserve, Hekou, Yunnan 661399, China Hekou Branch of Administration Bureau of Daweishan National Nature Reserve Honghe China; 4 University of Chinese Academy of Sciences, Beijing 10049, China University of Chinese Academy of Sciences Beijing China; 5 Royal Botanic Garden Edinburgh, 20A Inverleith Row, Edinburgh EH3 5LR, Scotland, UK Royal Botanic Garden Edinburgh Edinburgh United Kingdom

**Keywords:** *Amentotaxus×hybridia*, molecular evidence, natural hybridisation, new hybrid, Taxaceae

## Abstract

During floristic surveys of Taxaceae in Hekou County, Yunnan Province, China, a putative natural hybrid between *A.yunnanensis* H.L. Li and *A.hekouensis* L.M. Gao was collected. Morphological and molecular evidence confirms its status as a natural hybrid. Amentotaxus×hybridia L.M. Gao has linear or linear-lanceolate leaves 6–13 cm × 1.0–1.5 cm, white stomatal bands with 34–40 rows on abaxial side, 2.5–3.5 mm, slightly wider than leaf margins; 3–6 seeds borne at the base of the branchlet, peduncle 1.3–1.6 cm long with 3–4 rows of persistent basal bracts.

## ﻿Introduction

The genus *Amentotaxus* Pilg. (1916) in the family Taxaceae comprises five or six species (Fu et al. 1999; Farjon 2010; Yang et al. 2017). Southeast Yunnan (China) and adjacent areas of Vietnam and Laos are the centre of diversity for this genus with more than half of the known species recorded (Nguyen et al. 2004; Averyanov et al. 2014; Gao et al. 2019).

In February 2016, surveys were undertaken in the mountains near Nanxi town, Hekou county, Yunnan province, China to collect fertile material of the recently described species *Ametotaxushekouensis* L.M. Gao. This taxon was initially identified as a potential new species based on DNA barcoding data (Gao et al. 2017) and then subsequently formally described (Gao et al. 2019). During the 2016 surveys, a tree with vegetative morphology intermediate between *A.yunnanensis* H.L. Li and *A.hekouensis* but with seed cones resembling *A.hekouensis*, was found growing with several trees identified as either *A.yunnanensis* or *A.hekouensis*. Its intermediate characters indicated that it may represent a hybrid between *A.yunnanensis* and *A.hekouensis*. Initial sequencing of two DNA barcodes, the plastid DNA (ptDNA) *trnL-F* and the internal transcribed spacer of nuclear ribosomal DNA (nrITS), previously used for species identification within *Amentotaxus* (Gao et al. 2017) indicated that the paternally inherited (Collins et al. 2013) ptDNA*trnL-F* sequence was identical to that of *A.hekouensis* (GenBank accession number: KX059381). The ITS sequence of the hybrid individual (including the whole length of ITS1 and 5.8S, and a partial sequence of ITS2) was also identical to that of *A.hekouensis* (GenBank accession number: JF975885). However, we found many mixed nucleotide sites in the ITS sequence where they are polymorphic between *A.yunnanensis* and *A.hekouensis* ITS sequences. These sites had double peaks but with different heights in the Sanger sequencing trace file, which suggested the presence of interspecific hybridisation but required further research.

## ﻿Materials and methods

All measurements of the new hybrid of *Amentotaxus* were taken from dried herbarium specimens of the hybrid individual *GLM164267*. All measurements of *A.hekouensis* and *A.yunnanensis* were based on literature (Fu et al. 1999; Farjon 2010; Gao et al. 2019) and our collections and observations.

To confirm that the individual (*GLM164267*) is a hybrid between *A.hekouensis* and *A.yunnanensis*, we generated approximately 2.5GB (gigabase) of genome skimming data to assemble the complete plastid genome and nrITS region. The methods of DNA extraction, genome skimming sequencing, the plastome and nrITS sequences assembly, and gene annotation are detailed in Fu et al. (2022). We mapped the genome skimming reads back to the nrITS sequence to show the nucleotide composition of polymorphic sites of the hybrid individual along with the ITS sequence of *A.hekouensis* and *A.yunnanensis* (Gao et al. 2017).

## ﻿Results and discussion

The morphological trait comparison among the three taxa showed that several traits of the hybrid (Amentotaxus×hybridia), such as texture of leaves, width of stomatal bands, and number of rows of each stomatal band, are intermediate between *A.hekouensis* and *A.yunnanensis*, but with more similarity to *A.hekouensis*. The hybrid differs from its parental species by having linear or linear-lanceolate leaves, white stomatal bands with 34–40 rows that are slightly wider than the marginal bands in width; 3–6 seeds borne at the base of the branchlet, and 3–4 rows of persistent basal bracts at the peduncle (Table 1).

**Table 1.** Morphological character comparison of the three *Amentotaxus* taxa.

Characters	*A.hekouensis*	A.×hybridia	*A.yunnanensis*
Leaf Length (cm)	8–12.5 cm	6–13 cm	3.5–10 cm
Leaf width (cm)	9–14 mm	10–15 mm	8–12 mm
Leaf texture	thin, leathery	moderately thick, leathery	thick, leathery
Leaf apex	long acuminate	acuminate	obtuse or tapered
Width of stomatal bands	2.1–3.0 mm	2.5–3.5 mm	3–4 mm
No. rows of each stomatal band	25–30	34–40	c. 40
Ratio of stomatal band/marginal band	0.75–1	1.10–1.25	> 2
Marginal band colour in fresh leaves	bright green	green	yellowish green
No. of bract rows on peduncle	unknown	3–4	2

The nrITS sequence of the hybrid individual *de novo* was assembled using the GetOrganelle toolkit (Jin et al. 2020), which is identical to that of *A.hekouensis* (AM24). There are 6,891 clean reads mapped to the nrITS region with a mean sequencing coverage of 494×. The mapping reads of the ITS region showed that a total of 17 polymorphic sites of ITS sequence were consistent with the polymorphic nucleotide sites between *A.hekouensis* (AM24) and *A.yunnanensis* (AM21); 15 were point mutation and two mononucleotide indels (Table 2). The result confirmed that the individual is the result of interspecific hybridisation between *A.hekouensis* and *A.yunnanensis*. The dominant nucleotides of the hybrid individual are same as those of *A.hekouensis* (AM24), and the ratio of the polymorphic nucleotide sites between the parental species ranged from 1.7 to 3.2 with an average of 2.2 (Table 2). This closely corresponds to the results from the direct sequencing of ITS sequence for the hybrid individual by Sanger sequencing. The trace file showed double peaks with different height at the polymorphic sites, and resulted in a messy sequence for primer ITS4 trace file after the occurrence of an indel in the ITS2. These results also indicated that the hybrid may not represent an F1 generation of the hybrid.

**Table 2.** Sequence variation and polymorphisms of nrITS among the parent species and the hybrid.

nrITS	ITS1										ITS2						
Position	179	200	268	307	355	366	368	425	461	516	816	833	856	861	910	964	969
*A.yunnanensis* AM21 (female)	C	T	G	T	C	A	G	T	G	T	C	C	T	G	C	T	C
*A.hekouensis* AM24 (male)	T	C	A	C	T	T	T	C	C	C	T	–	C	A	G	–	G
A.×hybridia AM51	T/C	C/T	A/G	C/T	T/C	T/A	T/G	C/T	C/G	C/T	T/C	-/C	C/T	A/G	G/C	-/T	G/C
Nucleotide rate of polymorphic mapping sites#	2.0	2.2	2.2	2.3	2.5	2.0	2.0	2.1	2.2	2.3	3.2	2.6	2.6	2.1	2.0	1.9	1.7

^#^ The nucleotide rate is AM24/AM21 of the polymorphic mapping sites for the *A.×hybridia* individual (AM51) with genome skimming data.

The length of the plastome of the hybrid (*GLM164267*) is 137,786 bp with 35.8% GC content. A total of 198,855 reads were mapped to the assembled plastome with an average sequencing coverage of 215×. The whole plastome included 118 unique genes, comprising 4 rRNA genes, 31 tRNA genes (three tRNA have two copies: *trnI-CAU*, *trnN-GUU* and *trnQ-UUG*) and 83 protein-coding genes. The plastid genome phylogenetic tree of *Amentotaxus* showed that the hybrid individual fell in the clade of *A.hekouensis* (data not shown). As the plastid genome is paternally inherited in *Amentotaxus*, the results demonstrate that *A.hekouensis* is the paternal parent with *A.yunnanensis* as the maternal parent. In our previous DNA barcoding study (Gao et al. 2017), we also found a hybrid individual (Coll. No.: *P11120*) from Thai Phin Tung Commune Dong Van, Ha Giang Province, Vietnam, which, at the time, was the first evidence of an interspecific hybridisation in natural populations of *Amentotaxus*. In that case, the paternal species was *A.yunnanensis* rather than *A.hekouensis*, and the hybrid was morphologically more similar to the paternal species *A.yunnanensis*. Both results demonstrate natural hybridisation between *A.yunnanensis* and *A.hekouensis* when they occur sympatically. The hybrids are morphologically more similar to the paternal species. The results also indicate that bidirectional hybridisation does occur.

## ﻿Taxonomic treatment

### ﻿ Amentotaxus × hybridia

Taxon classificationPlantaeCupressalesTaxaceae

L.M.Gao & G.L.Zhang sp. nov.

644847B5-764C-523D-B2AC-75B35A37226E

urn:lsid:ipni.org:names:77319909-1

**Figure 1.**
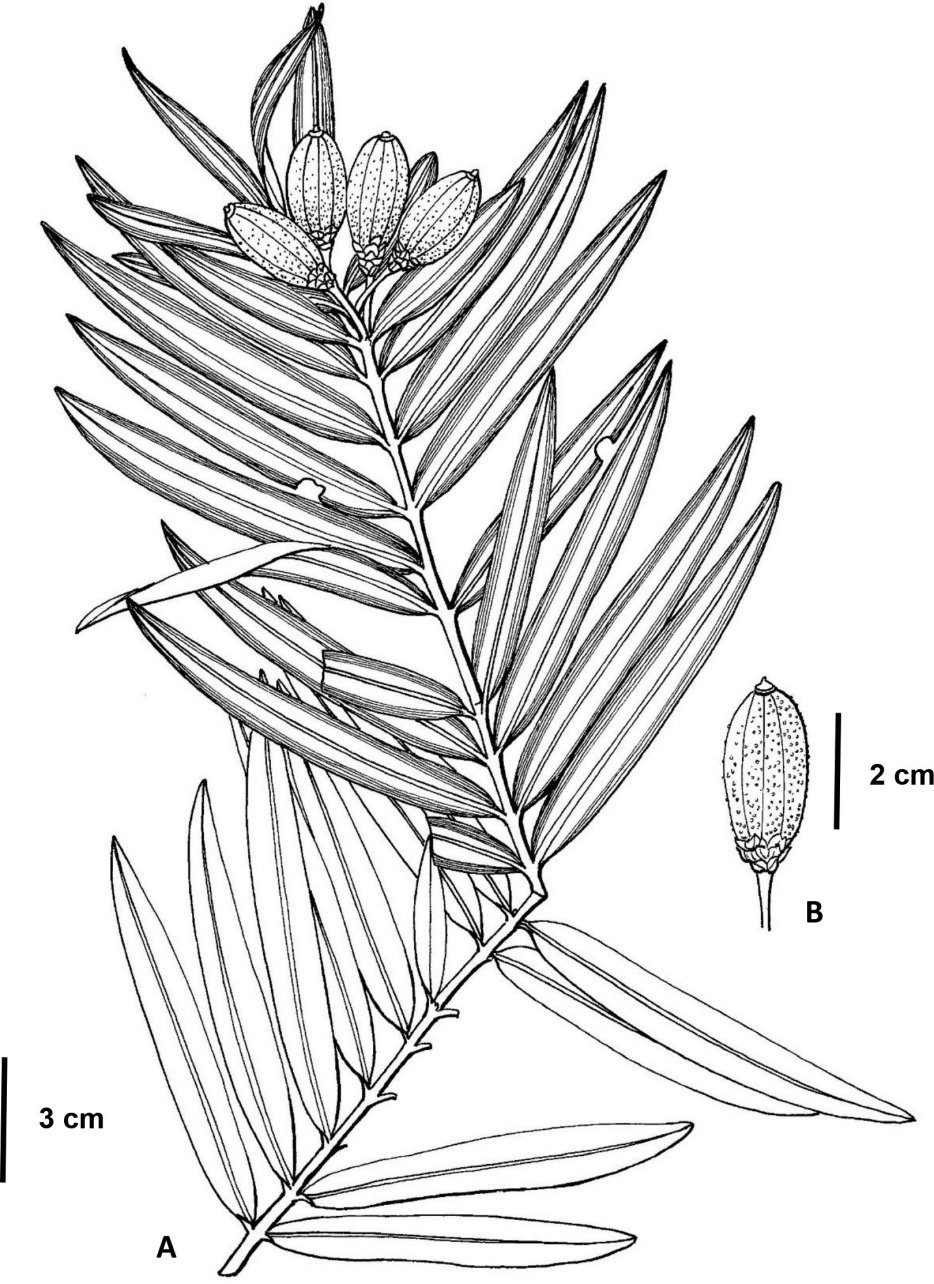
Amentotaxus×hybridia L.M. Gao & G.L. Zhang (from the holotype, drawn by Ling Wang) **A** branchlet with seeds **B** seed with peduncle and bracts.

**Figure 2.**
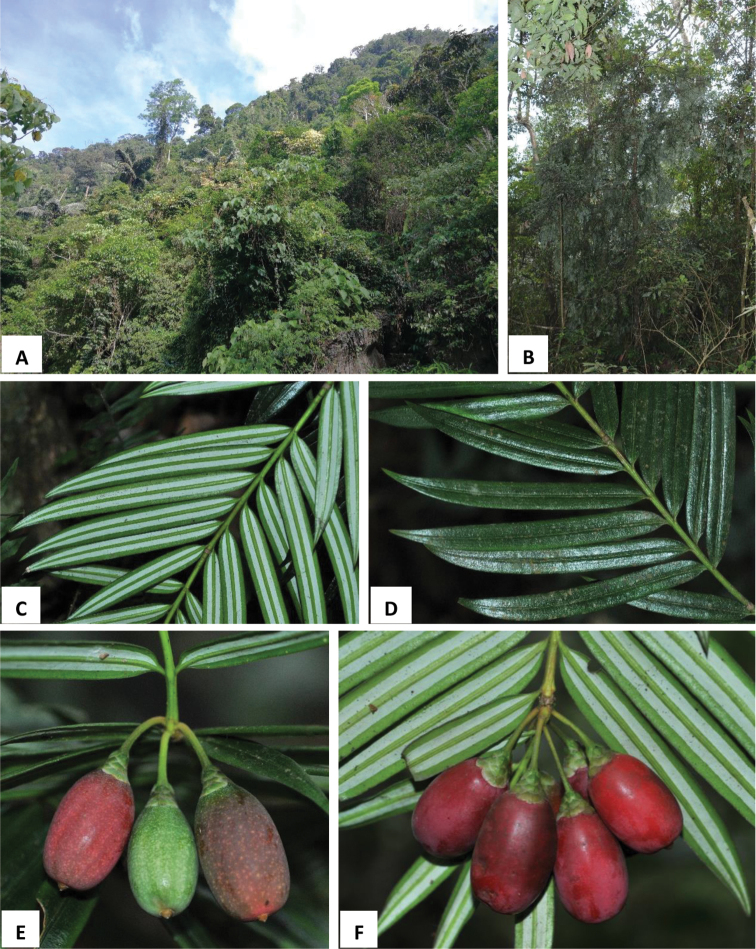
Amentotaxus×hybridia L.M. Gao & G.L. Zhang **A** habitat **B** habit **C** branchlet with abaxial leaves **D** branchlet with adaxial leaves **E** seeds bearing branchlet and seeds with mucronate apex **F** seed-bearing structure with peduncle and bracts.

Figs 1 , 2 , Table 1

#### Diagnosis.

Amentotaxus×hybridia L.M. Gao & G.L. Zhang resembles *A.hekouensis* L.M. Gao, but differs by its larger linear or linear-lanceolate leaves of 6–13 cm × 1.0–1.5 cm, stomatal bands with 34–40 rows on abaxial side, 2.5–3.5 mm wide, slightly wider than the green leaf margins; 3–6 seeds borne at the base of the branchlets, peduncle 1.3–1.6 cm long, 3–4 rows of persistent basal bracts (Table 1).

#### Type.

China. Yunnan: Qincaitang Mt., Longbao village, Nanxi Town, Hekou County, Honghe, 22°39'49"N, 104°01'17"E, elevation 1100 m, 15 February 2016 (with mature seeds), *Zhang GL*, *GLM164267* (Holotype: KUN, isotype: KUN).

#### Morphological description.

Small tree up to 5 m tall, bark brown gray; branch cylindric or subtetragonal, yellowish green; leafy branchlets ascending, broadly rectangular to oblong-elliptic in outline, axis green in 1^st^ year, greenish yellow in 2^nd^ and 3^rd^ years, quadrangular or subterete in cross section. Leaves borne at 40–70° to the branchlet axis, distichous, twisted at the short petiolate or nearly sessile base, subsessile or petiolate, petiole to 2 mm long, almost opposite, 5–7 leaf pairs on each branchlet; Leaves leathery, linear or linear-lanceolate, 6–13 cm × 1.0–1.5 cm, straight, sometimes slightly falcate, cuneate at base, asymmetric, apex acuminate, leaf margin narrowly revolute, sometime flat; leaf marginal band green when fresh, yellowish green when dry, 2.0–3.0 mm wide; stomatal bands white when fresh and yellow white when dry, 2.5–3.5 mm wide, slightly wider than green leaf margins, stomata in 34–40 rows of each band, densely arranged; midvein slightly sunken or flat adaxially, raised abaxially, 1.5–2.0 mm wide, narrower than the stomatal and marginal bands, yellowish green, same color as the branchlet. Seed-bearing structures in clusters of 3–6 at the base of the branchlet, not from subtending leaf. Aril reddish purple when ripe. Mature seed reddish purple, obovoid-ellipsoid, 2.5–3.0 cm × 1.4–1.6 cm, mucronate at apex, naked; peduncle 1.3–1.6 cm long, compressed-quadrangular, slightly dilated below bracts; 3–4 rows of persistent basal bracts on peduncle, four bracts per row, imbricate, obovate or obovate-oblong, with a ridge in middle. Seed maturity February. Pollen cone not seen.

#### Distribution and ecology.

Amentotaxus×hybridia has only been found in the karst montane monsoon evergreen forest in southeast Yunnan province along the border between China (Hekou, Yunnan) and Vietnam occurring at an elevation around 1100 m.

#### Phenology.

Seed matures in February.

#### Etymology.

The specific epithet is derived from the natural hybridisation between *A.yunnanensis* and *A.hekouensis*.

#### Conservation status.

As a natural hybrid, Amentotaxus×hybridia is not eligible for listing under the current IUCN Categories and Criteria (IUCN Standards and Petitions Committee 2022). However, as it occurs in areas where *A.yunnanensis* and *A.hekouensis* are sympatric, hybridisation could lead to a loss of genetic diversity for those species. In addition, the lack of a reproductive barrier between *A.hekouensis* and *A.yunnanensis* has implications for ex-situ conservation programs, especially if collections are intended to be used as a source of material for reintroduction or for long term conservation in cultivation.

## Supplementary Material

XML Treatment for Amentotaxus × hybridia
